# Distributed State Estimation Using a Modified Partitioned Moving Horizon Strategy for Power Systems

**DOI:** 10.3390/s17102310

**Published:** 2017-10-11

**Authors:** Tengpeng Chen, Yi Shyh Eddy Foo, K.V. Ling, Xuebing Chen

**Affiliations:** School of Electrical and Electronic Engineering, Nanyang Technological University, Singapore 639798, Singapore; EDDYFOO@ntu.edu.sg (Y.S.E.F.); EKVLING@ntu.edu.sg (K.V.L.); CHEN0971@e.ntu.edu.sg (X.C.)

**Keywords:** distributed state estimation, moving horizon estimation, wide-area monitoring, sensor measurement, power systems, outliers

## Abstract

In this paper, a distributed state estimation method based on moving horizon estimation (MHE) is proposed for the large-scale power system state estimation. The proposed method partitions the power systems into several local areas with non-overlapping states. Unlike the centralized approach where all measurements are sent to a processing center, the proposed method distributes the state estimation task to the local processing centers where local measurements are collected. Inspired by the partitioned moving horizon estimation (PMHE) algorithm, each local area solves a smaller optimization problem to estimate its own local states by using local measurements and estimated results from its neighboring areas. In contrast with PMHE, the error from the process model is ignored in our method. The proposed modified PMHE (mPMHE) approach can also take constraints on states into account during the optimization process such that the influence of the outliers can be further mitigated. Simulation results on the IEEE 14-bus and 118-bus systems verify that our method achieves comparable state estimation accuracy but with a significant reduction in the overall computation load.

## 1. Introduction

Power system state estimation (PSSE) plays an indispensable part in the power industry [1]. One common centralized approach named the weighted least squares (WLS) has been widely used for PSSE, employing a nonlinear measurement model. In recent years, phasor measurement units (PMUs) have drawn much attention because they can provide voltage and current phasors and the measurement model becomes linear [2,3]. The computation complexity will become simpler.

Even though PMUs can result in measurements with higher accuracy, the PSSE still constitutes a major challenge due to the presence of bad data or outliers [3,4,5]. Such outliers that are far away from the expected measuring data create the potential risk of misleading the estimated result [6]. The WLS, primarily based on a single snap shot of measurements, is not a robust method and it may lead to a biased estimated result even when a single bad measurement occurs [3,7]. One common way to alleviate the influence of outliers is to use more snap shots of measurements. Moreover, in order to reduce the influence of outliers further, the largest normalized residuals (LNR) test [8] is usually used in WLS to deal with bad data. Many other centralized algorithms based on the WLS have been proposed in [9,10,11]. Due to the increasing number of PMUs installed in substations, it is reasonable to assume that the power systems will be only measured by PMUs in the near future [12]. The authors in [13] use an adaptive approach in updating the accuracies of the PMUs while employing local decision metrics and an Internet of Things (IoT) paradigm. It allows considering adaptive values of the accuracies of different measurement devices in the estimation process. However, the algorithm is basically solving a centralized state estimation problem for the distribution system. In [3,4], the least absolute value (LAV) has been proposed to handle the outliers where the measurements are collected from PMUs. However, the algorithms described so far are mostly based on the measurement model only. A robust estimator via the moving horizon strategy has been developed for PSSE in [14]. The re-weighted moving horizon estimation (MHE) aims to solve an optimization problem at each time instant by using a limited amount of the most recent information, and the objective function of re-weighted MHE includes the measurement model error, the process model error and the error in the state estimate at the beginning of the window. Moreover, the constraints on states have also been exploited. By having these constraints in the optimization process, it is more robust to the outliers. The price to pay is an increased complexity since the computational load will increase. A modified MHE (mMHE) has been developed in [15]. In this reference, the objective function of the mMHE consists only of the sensor model error and the error from the prior estimate. The state at the beginning of the window is obtained in the optimization, instead of all states in the window under the scheme of MHE. Therefore, the mMHE will have a higher computing speed than the MHE. The tradeoff is the estimated accuracy of mMHE is a little smaller than that of MHE, but their difference is insignificant.

Due to the fact that power systems become larger and increase in complexity, the centralized estimators that process the measurements from the whole grid may no longer be feasible [16,17]. Based on the rapid growth of usage of the wide-area monitoring systems for modern power grids, many approaches on distributed state estimation are proposed. A fully distributed state estimation approach is presented whereby each local area solves the system-wide states [18]. The authors in [9] propose a fully decentralized adaptive re-weighted state estimation algorithm for hybrid PSSE, where both measurements from the supervisory control and data acquisition (SCADA) system and PMUs are used. However, for the two algorithms mentioned above, a large amount of iterations are required when the power systems are large. A distributed state estimation for power systems with linear models has been proposed and the alternating direction method of multipliers (ADMM) is used to solve the optimization problem [19]. A distributed robust bilinear state estimation method is proposed to multi-area power systems with nonlinear measurements where interregional communication is required but a central coordinator is not necessary [20]. One drawback of the distributed algorithms described so far is that they are largely based on just the measurement model and fall into the WLS category. The authors in [21] propose a new distributed framework where the process model is used. The average consensus algorithm is applied such that the substations can maintain the global state through information exchange with neighbors. However, for all the approaches mentioned above, the constraints on states are not considered during the optimization process. This may lead to a suboptimal solution. A distributed moving horizon estimation (DMHE) for power systems has been proposed in [22]. It is suitable for advanced applications such as wide-area monitoring and control that require the system-wide states to be available to all the regional transmission organizations (RTOs) [23]. However, the computational load for each local area is still heavy. The authors in [24] propose a distributed state estimation method for linear systems, and it is known as the partitioned moving horizon estimation (PMHE). The PMHE is more reasonable and suitable for large-scale systems monitoring because each local area (or subsystem) solves for the local states via a smaller optimization problem and the computational load is smaller whereby measurements are only sent to the local estimator but not to the centralized estimator, so a large amount of communication burden would be saved. Meanwhile, information is exchanged among neighboring areas only so the communication load is also small.

In this paper, considering the high speed of mMHE, the accuracy of MHE, and the advantage of PMHE to implement the MHE in a distributed way, a distributed state estimation method named the modified PMHE (mPMHE) is proposed and implemented for PSSE. Under the scheme of mPMHE, the error from the process model is ignored in the objective function. The proposed mPMHE has the following advantages:

- Instead of all the states in the window estimated by PMHE, the mPMHE only estimates the state vector at the beginning of the window so it is faster than PMHE. The estimated precision of mPMHE is slightly lower than that of PMHE, but their difference is insignificant. The mPMHE achieves comparable state estimation accuracy but with a significant reduction in the overall computation load.
- It is a distributed algorithm and is suitable for large-scale PSSE. Each local area solves for its own local states by using the local measurements and the estimated results from neighboring areas, so the computation load is small. In addition, the communication load is also small because the information is exchanged among neighboring areas only.
- Constraints are taken into account during the optimization process and it is robust to outliers. Hence, good estimated results could be obtained.

This paper is organized as follows. The centralized state estimation is briefed in Section 2 and the mPMHE approach is proposed in Section 3. The simulations on the IEEE 14-bus and 118-bus systems are shown in Section 4 and conclusions are made in Section 5 respectively.

## 2. Centralized State Estimation

### 2.1. Measurement Model and State Equation

The linear measurement model based on the PMUs [3] is given as

(1) $$\begin{array}{ccc} z_{k} & = & Hx_{k}+v_{k}, \end{array}$$

where *H* is the measurement matrix and *k* is the time step. This paper uses rectangular coordinates. $z\in R^{m}$ is the measurement vector composed of the real and imaginary components of the bus voltage (or the line current) phasors. The state vector $x\in R^{n}$ includes the real and imaginary parts of the voltage phasors. $v\in R^{m}$ is assumed to be noise with covariance *R*. The standard deviation of *i*-th measurement noise is denoted as $\sigma_{i}$. In this paper, the following assumption is held:

**Assumption** **1.** The power system is observable so the matrix $G=H^{T}H$ is full rank.

The following simplified process model is considered for the state estimation [25,26]:

(2) $$\begin{array}{c} x_{k+1}=Ax_{k}+w_{k}, \end{array}$$

where *A* is assumed to be an identity matrix according to [26] and $w_{k}$ represents the zero-mean disturbance with covariance *Q*.

### 2.2. Weighted Least Squares (WLS)

The WLS is an iterative algorithm that is applied to measurements including the power injections and power flows are usually collected from the SCADA system. However, the iterations are not necessary when PMUs are used. A traditional power system may be considered as a quasi-static system [25] because load demands change slowly and hence the state changes slowly. The sampling time of PMU measurements is usually in the order of milliseconds while the estimates are usually updated once every few minutes if the measurements are collected from SCADA [27]. In order to alleviate the influence of bad measurements, a total horizon length of N+1 PMU measurements are used. During this interval, it is assumed that the system state is constant. The state $x_{t}$ can be estimated by solving the following cost function:

(3) $$\begin{array}{c} \min_{{\hat{x}}_{t}}J=\min_{{\hat{x}}_{t}}\sum_{i=1}^{m}\sum_{k=t-N}^{t}\rho \left(e_{i,k}\right)=\min_{{\hat{x}}_{t}}\sum_{i=1}^{m}\sum_{k=t-N}^{t}\frac{{\left(e_{i,k}\right)}^{2}}{2\sigma_{i}^{2}} \end{array}$$

where $e_{i,k}$ is the *i*-th measurement residual at time step *k*,

(4) $$e_{i,k}=z_{i,k}-H_{i}{\hat{x}}_{k}$$

Equation (4) gives $\frac{\partial e_{i,t}}{\partial {\hat{x}}_{t}}=-{\left(H_{i}\right)}^{T}$. Differentiating the above cost function (3) with respect to ${\hat{x}}_{t}$,

(5) $$\begin{array}{ccc} \frac{\partial J}{\partial {\hat{x}}_{t}} & = & \frac{\partial J}{\partial e_{i,t}}\frac{\partial e_{i,t}}{\partial {\hat{x}}_{t}}=\sum_{i=1}^{m}\sum_{k=t-N}^{t}\frac{\partial \rho (e_{i,k})}{\partial e_{i,k}}\frac{1}{e_{i,k}}e_{i,k}\frac{\partial e_{i,k}}{\partial {\hat{x}}_{t}}\\ & = & -\sum_{i=1}^{m}\sum_{k=t-N}^{t}W_{i,k}e_{i,k}H_{i}^{T} \end{array}$$

where

$$W_{i,k}=\frac{1}{\sigma_{i}^{2}}$$

Using (4), Equation (5) can be written as

(6) $$\begin{array}{ccc} \frac{\partial J}{\partial {\hat{x}}_{t}} & = & -\sum_{i=1}^{m}\sum_{k=t-N}^{t}W_{i,k}\left(z_{i,k}-H_{i}{\hat{x}}_{t}\right)H_{i}^{T}\\ & = & -{\bar{H}}^{T}W\left(Z-\bar{H}{\hat{x}}_{t}\right)\\ & = & -{\bar{H}}^{T}WE\\ & ≜ & \Psi (E) \end{array}$$

where

$$\begin{array}{ccc} \bar{H} & = & {\left[\begin{array}{c} H^{T}\cdots H^{T} \end{array}\right]}^{T}\in R^{((N+1)m)\times n}\\ Z & = & {\left[\begin{array}{c} z_{t-N}^{T}\cdots z_{t}^{T} \end{array}\right]}^{T}\in R^{\left((N+1)m\right)}\\ E & = & {\left[\begin{array}{c} e_{t-N}^{T}\cdots e_{t-N}^{T} \end{array}\right]}^{T}\in R^{\left((N+1)m\right)}\\ W & = & \mathrm{diag}\left(\frac{1}{\sigma_{1}^{2}},\ldots ,\frac{1}{\sigma_{m}^{2}},\ldots ,\frac{1}{\sigma_{1}^{2}},\ldots ,\frac{1}{\sigma_{m}^{2}}\right)\in R^{\left((N+1)m)\times ((N+1)m\right)} \end{array}$$

Under Assumption 1, the matrix ${\bar{H}}^{T}W\bar{H}$ is also full rank and is an invertible matrix. Next, set Ψ(E)=0, then from (6) the estimation of $x_{t}$ is given by

(7) $$\begin{array}{ccc} {\hat{x}}_{t} & = & {\left({\bar{H}}^{T}W\bar{H}\right)}^{-1}{\bar{H}}^{T}WZ \end{array}$$

Note that the WLS is not a robust estimator and the largest normalized residuals (LNR) method [8] is usually used in WLS to deal with bad data. The normalized residuals are calculated as follows:

$$\begin{array}{ccc} \bar{R} & = & \mathrm{diag}(\sigma_{1}^{2},\ldots ,\sigma_{m}^{2},\ldots ,\sigma_{1}^{2},\ldots ,\sigma_{m}^{2})\\ \bar{G} & = & {\bar{H}}^{T}{\bar{R}}^{-1}\bar{H}\\ \bar{\Omega } & = & \bar{R}-\bar{H}{\bar{G}}^{-1}{\bar{H}}^{T}\\ e_{i,k}^{norm} & = & \frac{|e_{i,k}|}{\sqrt{{\bar{\Omega }}_{ii}}} \end{array}$$

The normalized residuals $e_{i,k}^{norm}$ are calculated according to the residual covariance matrix $\bar{\Omega }$ and measurement residual $e_{i,k}$. If the normalized residuals $e_{i,k}^{norm}$ are larger than a pre-determined threshold, the largest one will correspond to the bad measurement $Z_{i}^{bad}$. Once the largest normalized residual is found, the corresponding measurement is updated:

$$\begin{array}{c} Z_{i}^{new}=Z_{i}^{bad}-\frac{{\bar{R}}_{ii}}{{\bar{\Omega }}_{ii}}e_{i,k}^{bad} \end{array}$$

The states will then be recalculated based on the updated measurements $Z_{i}^{new}$. Several iterations may be needed in order to make sure that all normalized residuals are less than the pre-determined threshold, for example, 3.0 [3].

### 2.3. Moving Horizon Estimation (MHE)

The common WLS estimator for power system state estimation has been discussed in previous subsection. However, the constraints on the states are not considered and this may lead to suboptimal estimates. In this subsection, the MHE algorithm in [28] is applied to the PSSE problem.

(8) $$\begin{array}{c} \Theta_{t}^{*}=\min_{{\hat{x}}_{t-N},\ldots ,{\hat{x}}_{t}}\Psi_{t}\left({\hat{x}}_{t-N},\ldots ,{\hat{x}}_{t}\right) \end{array}$$

subject to

$$\begin{array}{c} {\hat{x}}_{k+1}=A{\hat{x}}_{k}+{\hat{w}}_{k},\;k=t-N,\ldots ,t-1\\ z_{k}=H{\hat{x}}_{k}+{\hat{v}}_{k},\;k=t-N,\ldots ,t\\ {\hat{x}}_{k}\in X \end{array}$$

where X is a set of constraints defined by linear inequalities. In the following, the notation t−N|t−N−1 refers to the time step for prediction from step t−N−1 to t−N. Denote ∥·∥ as the Euclidean norm of a vector and ${∥\cdot ∥}_{S}^{2}$ as the square of the weighted Euclidean norm of a vector, ${∥x∥}_{S}^{2}=x^{T}Sx$, where *S* is a positive definite matrix.

The objective function of MHE in (8) at time step *t* is given by

(9) $$\begin{array}{ccc} \Psi_{t}\left({\hat{x}}_{t-N},\ldots ,{\hat{x}}_{t}\right) & = & \frac{1}{2}\sum_{k=t-N}^{t}∥{\hat{v}}_{k}{∥}_{R^{-1}}^{2}+\frac{1}{2}\sum_{k=t-N}^{t-1}{∥{\hat{w}}_{k}∥}_{Q^{-1}}^{2}+\Phi_{t-N} \end{array}$$

where the arrival cost $\Phi_{t-N}$ is given as

(10) $$\begin{array}{c} \Phi_{t-N}=\frac{1}{2}{∥{\hat{x}}_{t-N}-{\hat{x}}_{t-N|t-N-1}∥}_{P_{t-N|t-N-1}^{-1}}^{2} \end{array}$$

For the arrival cost (10), we calculate $P_{t-N|t-N-1}$ from $P_{t-N-1|t-N-2}$ using the equation derived in [29]:

(11) $$\begin{array}{ccc} P_{t-N|t-N-1} & = & AP_{t-N-1|t-N-2}A^{T}-AP_{t-N-1|t-N-2}H^{T}{\left(R+HP_{t-N-1|t-N-2}H^{T}\right)}^{-1}\\ & & \times HP_{t-N-1|t-N-2}A^{T}+Q \end{array}$$

The objective function of MHE includes three error terms: (i) the error between the measurement and sensor model prediction by (2); (ii) the error between the estimated state and its process model prediction by (1); (iii) and the error between the initial state ${\hat{x}}_{t-N}$ in the horizon and the *a priori* state estimate ${\hat{x}}_{t-N|t-N-1}$.

### 2.4. Modified Moving Horizon Estimation (mMHE)

In this subsection, we briefly review the mMHE method presented in [15]. The mMHE is an approach that compromises between the computational complexity and the estimated accuracy. The objective function for mMHE includes two terms, a prior term given in (10) and the measurement error term given in (3),

(12) $$\begin{array}{c} \Theta_{t}^{*}=\min_{{\hat{x}}_{t-N}}\Psi_{t}\left({\hat{x}}_{t-N}\right) \end{array}$$

where

(13) $$\begin{array}{ccc} \Psi_{t}\left({\hat{x}}_{t-N}\right) & = & \frac{1}{2}\sum_{k=t-N}^{t}∥{\hat{v}}_{k}{∥}_{{\left(R\right)}^{-1}}^{2}+\frac{1}{2}{∥{\hat{x}}_{t-N}-{\hat{x}}_{t-N|t-N-1}∥}_{P_{t-N|t-N-1}^{-1}}^{2} \end{array}$$

Note that the MHE approach needs to solve the problem with a higher dimension (N+1)n and also acquires all the states in the current window, ${\hat{x}}_{t-N},\ldots ,{\hat{x}}_{t}$. The objective function of WLS given in (3) only includes the first error term of MHE and its dimension is *n*. The mMHE solves a problem with the same dimension as (3) so the computational time is also faster than MHE. Even though the errors from the process model are not included in the objective function of mMHE, there still exists some uncertainty and *Q* should be combined in (11).

Driven by the increasing demand of wide-area system monitoring and the high speed of mMHE, a distributed approach named the modified partitioned MHE (mPMHE) is proposed in the next section.

## 3. Modified Partitioned Moving Horizon Estimation (mPMHE)

In the previous section, the WLS, MHE and mMHE are implemented under the centralized setup, where all PMU measurements are collected and then sent to a control center. However, it may not be feasible in practice when a power grid is large-scale. In this section, on the basis of PMHE proposed in [24], we develop a distributed state estimation approach named the modified PMHE (mPMHE) for large-scale power system monitoring, where each local area estimates its local states based on local measurements and information exchanges among the neighboring areas. Moreover, the mPMHE takes less time than PMHE. The tradeoff is a decrease in the estimated accuracy. The convergence of mPMHE follows that of PMHE presented in [24].

### 3.1. mPMHE Problem Formulation

Let models (1) and (2) be partitioned into *ℓ* control areas with non-overlapping states [24]:

(14) $$\begin{array}{cc} x_{t+1}^{\left[i\right]}= & A^{\left[i\right]}x_{t}^{\left[i\right]}+{\tilde{A}}^{\left[i\right]}x_{t}+w_{t}^{\left[i\right]} \end{array}$$

(15) $$\begin{array}{cc} z_{t}^{\left[i\right]}= & H^{\left[i\right]}x_{t}^{\left[i\right]}+{\tilde{H}}^{\left[i\right]}x_{t}+v_{t}^{\left[i\right]} \end{array}$$

where $x_{t}^{\left[i\right]}\in R^{n_{i}}$ is the local states in area *i*, $z_{t}^{\left[i\right]}\in R^{m_{i}}$ represents the local measurements, $H^{\left[i\right]}\in R^{m_{i}\times n_{i}}$ is the local measurement matrix, $w_{t}^{\left[i\right]}\in R^{n_{i}}$ and $v_{t}^{\left[i\right]}\in R^{m_{i}}$ are the noise with covariance $Q^{\left[i\right]}$ and $R^{\left[i\right]}=\mathrm{diag}\left(\sigma_{1}^{2},\ldots ,\sigma_{m_{i}}^{2}\right)$, respectively. Matrices $\tilde{A}$ and $\tilde{H}$ have structures in the form of

(16) $$\begin{array}{ccc} \tilde{A} & = & A-A^{*}=\left[\left({\tilde{A}}^{\left[1\right]}\right){}^{T}\ldots \left({\tilde{A}}^{\left[\ell \right]}\right){}^{T}\right]{}^{T} \end{array}$$

(17) $$\begin{array}{ccc} \tilde{H} & = & H-H^{*}=\left[\left({\tilde{H}}^{\left[1\right]}\right){}^{T}\ldots \left({\tilde{H}}^{\left[\ell \right]}\right){}^{T}\right]{}^{T} \end{array}$$

in which $A^{*}=\mathrm{diag}\left(A^{\left[1\right]},\ldots ,A^{\left[\ell \right]}\right)$ and $H^{*}=\mathrm{diag}\left(H^{\left[1\right]},\ldots ,H^{\left[\ell \right]}\right)$. The global vectors are $x_{t}={\left[{\left(x_{t}^{\left[1\right]}\right)}^{T}\ldots {\left(x_{t}^{\left[\ell \right]}\right)}^{T}\right]}^{T}$ and $z_{t}={\left[{\left(z_{t}^{\left[1\right]}\right)}^{T}\ldots {\left(z_{t}^{\left[\ell \right]}\right)}^{T}\right]}^{T}$.

We assume that the power system partitioning is based on the following assumption:

**Assumption** **2.** The pair $\left(A^{\left[i\right]},H^{\left[i\right]}\right)$ is locally observable, for i=1,…,ℓ.

Denote ${\hat{x}}_{k|t-1}^{\left[i\right]}$ as the estimation of $x_{k}^{\left[i\right]}$ performed at time step t−1. we approximate the covariance of $\left(x_{k|t-1}-{\hat{x}}_{k|t-1}\right)$ as $\Pi_{k|t-1}=\mathrm{diag}\left(\Pi_{k|t-1}^{\left[1\right]},\ldots ,\Pi_{k|t-1}^{\left[\ell \right]}\right)$.

In this paper, the proposed mPMHE-i at time *t* is defined as:

(18) $$\begin{array}{c} \Theta_{t}^{*[i]}=\min_{{\hat{x}}_{t-N}^{\left[i\right]}}\Psi_{t}\left({\hat{x}}_{t-N}^{\left[i\right]}\right) \end{array}$$

subject to

$$\begin{array}{ccc} {\hat{x}}_{k+1}^{\left[i\right]} & = & A^{\left[i\right]}{\hat{x}}_{k}^{\left[i\right]}+{\hat{w}}_{k}^{\left[i\right]},k=t-N,\ldots ,t-1\;\\ z_{k}^{\left[i\right]} & = & H^{\left[i\right]}{\hat{x}}_{k}^{\left[i\right]}+{\tilde{H}}^{\left[i\right]}{\hat{x}}_{k|t-1}+{\hat{v}}_{k}^{\left[i\right]},k=t-N,\ldots ,t\\ {\hat{x}}_{k}^{\left[i\right]} & \in & X^{i} \end{array}$$

where $X^{i}$ is the constraint set.

The local cost function in (18) is given by

(19) $$\begin{array}{ccc} \Psi_{t}\left({\hat{x}}_{t-N}^{\left[i\right]}\right) & = & \frac{1}{2}\sum_{k=t-N}^{t}∥{\hat{v}}_{k}^{\left[i\right]}{∥}_{{\left(R_{k|t-1}^{\left[i\right]}\right)}^{-1}}^{2}+\frac{1}{2}{∥{\hat{x}}_{t-N}^{\left[i\right]}-{\hat{x}}_{t-N|t-1}^{\left[i\right]}∥}_{{\left(\Pi_{t-N|t-1}^{\left[i\right]}\right)}^{-1}}^{2} \end{array}$$

in which

(20) $$\begin{array}{ccc} R_{k|t-1}^{\left[i\right]} & = & R^{\left[i\right]}+{\tilde{H}}^{\left[i\right]}\Pi_{k|t-1}\left({\tilde{H}}^{\left[i\right]}\right){}^{T} \end{array}$$

where $\tilde{H}$ is quite sparse and the second term on the right-hand side of (20) depends only on the neighboring areas.

### 3.2. Update Matrices $\Pi_{t-N|t-1}^{\left[i\right]}$

Denote the local observability matrix as $O_{N}^{\left[i\right]}=\left[{\left(H^{\left[i\right]}\right)}^{T}\ldots \left(H^{\left[i\right]}{\left(A^{\left[i\right]}\right)}^{N-1}\right){}^{T}\right]{}^{T}$ and 0 as the matrix of zero elements. We follow the method given by [24] to update $\Pi_{t-N|t-1}^{\left[i\right]}$ via the following equation:

$$\begin{array}{ccc} \Pi_{t-N|t-1}^{\left[i\right]} & = & A^{\left[i\right]}{\bar{\Pi }}_{t-N-1|t-2}^{\left[i\right]}{\left(A^{\left[i\right]}\right)}^{T}+Q_{t-N-1|t-2}^{\left[i\right]}-A^{\left[i\right]}{\bar{\Pi }}_{t-N-1|t-2}^{\left[i\right]}{\left(O_{N}^{\left[i\right]}\right)}^{T}\\ & & \times {\left(O_{N}^{\left[i\right]}{\bar{\Pi }}_{t-N-1|t-2}^{\left[i\right]}{\left(O_{N}^{\left[i\right]}\right)}^{T}+{\tilde{R}}_{N|t-2}^{\left[i\right]}\right)}^{-1}O_{N}^{\left[i\right]}{\bar{\Pi }}_{t-N-1|t-2}^{\left[i\right]}{\left(A^{\left[i\right]}\right)}^{T} \end{array}$$

where

$$\begin{array}{ccc} {\bar{\Pi }}_{t-N-1|t-2}^{\left[i\right]} & = & \left(\left(\Pi_{t-N-1|t-2}^{\left[i\right]}\right){}^{-1}+{\left(H^{\left[i\right]}\right)}^{T}\left(R_{t-N-1|t-2}^{\left[i\right]}\right){}^{-1}H^{\left[i\right]}\right){}^{-1},\\ {\tilde{R}}_{N|t-2}^{\left[i\right]} & = & R_{N|t-2}^{\left[i\right]}+L_{w,N}^{\left[i\right]}Q_{N-1|t-2}^{\left[i\right]}{\left(L_{w,N}^{\left[i\right]}\right)}^{T},\\ R_{N|t-2}^{\left[i\right]} & = & \mathrm{diag}(R_{t-N|t-2}^{\left[i\right]},\ldots ,R_{t-1|t-2}^{\left[i\right]}),\\ Q_{N-1|t-2}^{\left[i\right]} & = & \mathrm{diag}(Q_{t-N|t-2}^{\left[i\right]},\ldots ,Q_{t-2|t-2}^{\left[i\right]}),\\ L_{w,N}^{\left[i\right]} & = & \left[\begin{array}{cccc} 0 & 0 & \ldots & 0\\ H^{\left[i\right]} & 0 & \ldots & 0\\ \vdots & \vdots & \ddots & \vdots \\ H^{\left[i\right]}{\left(A^{\left[i\right]}\right)}^{N-2} & H^{\left[i\right]}{\left(A^{\left[i\right]}\right)}^{N-3} & \ldots & H^{\left[i\right]} \end{array}\right]. \end{array}$$

## 4. Simulation Results

In this section, simulations on the IEEE 14-bus and 118-bus systems using the mMHE and mPMHE algorithms will be presented. We also illustrate the effect of the number of PMUs installed in the power systems and consider two scenarios: one with redundant observations on selected buses and one with a minimum number of PMUs for full topological observation.

### 4.1. Simulations on the IEEE 14-Bus System

#### 4.1.1. Redundant Observations

**Figure 1:**
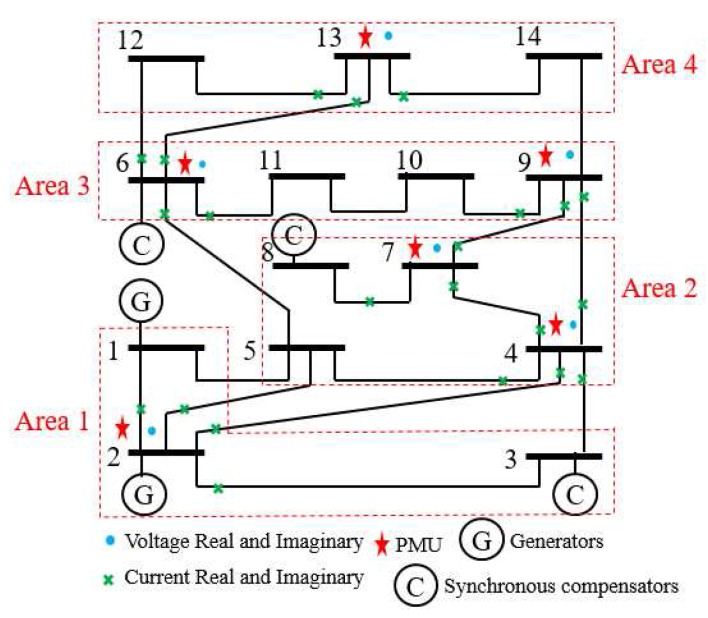
IEEE 14-bus system with phasor measurement units (PMUs) .

**Figure 2:**
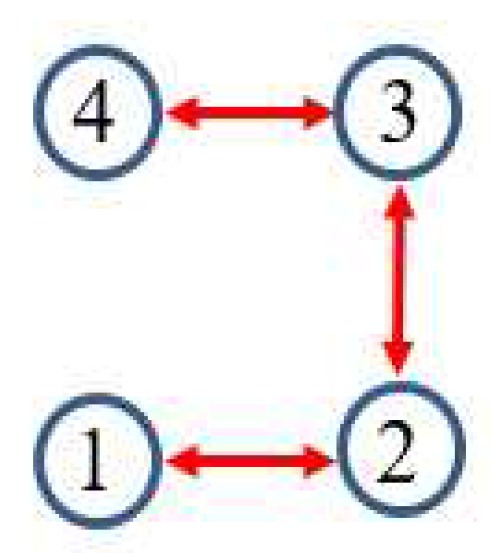
Communication scheme related to the partitioned type.

In this example, the IEEE 14-bus system is shown in Figure 1 where the PMUs are placed according to [26]. Fifty-eight measurements, $z_{i}$, i=1,⋯,58, consisting of 12 voltages (i=1,…,12) and 46 currents (i=13,…,58) are taken at each time instant *k*. When the horizon length N+1 increases, the computation load of MHE and PMHE will also increase due to the increasing dimension of the optimization problem arising from MHE and PMHE. However, the level of estimation accuracy will be higher when the horizon increases. It should be noted that the horizon length can be chosen according to the required estimation accuracy. In this paper, for simplicity, the measurements with horizon length 3, i.e., k=t−2,t−1,t are used to give one set of estimates. According to [1], the WLS estimator usually uses one snap shot of measurements (the horizon length is 1) to estimate, in this paper we also show the result for comparison and it is represented by “WLS(1)” in the following figures. The measurement matrix *H* is calculated according to the parameters in [30]. There are n=28 states in the vector $x={\left[V_{1}^{r}\;V_{2}^{r}\cdots V_{14}^{r}\;V_{1}^{im}\cdots V_{14}^{im}\right]}^{T}$ of (1) in which $V_{i}^{r}$ and $V_{i}^{im}$ ($j=1,\ldots ,\frac{n}{2}$) are the real and imaginary parts of the bus voltage phasors, respectively. *A* is simplified as an identity matrix according to [25,26]. The IEEE 14-bus system is partitioned into four non-overlapping areas where each local area is locally observable. Due to the partitioned type, information is only exchanged with its immediate neighbors and the communication scheme is given in Figure 2. The measurements allocated for each area are given in Table 1, where $I_{i,j}^{r}$ and $I_{i,j}^{im}$ define the real and imaginary components of current phasors from bus *i* to bus *j* respectively.

Define the Mean Square Error (MSE) in local area *i* for the estimation results at time step *k* as [3]

$$\begin{array}{c} MSE_{k}^{\left[i\right]}=\sqrt{\frac{1}{n_{i}}∥{\hat{x}}_{k|k}^{\left[i\right]}-x_{k}^{\left[i\right]}∥{}^{2}},\;i=1,\ldots ,\ell \end{array}$$

and denote the Average of Mean Square Error (AMSE) in local area *i* for $k\in [t_{c},\;t]$ to evaluate the estimation accuracy:

$$\begin{array}{c} {\mathrm{AMSE}}^{\left[i\right]}=\frac{1}{t-t_{c}+1}\sum_{k=t_{c}}^{t}\sqrt{\frac{1}{n_{i}}∥{\hat{x}}_{k|k}^{\left[i\right]}-x_{k}^{\left[i\right]}∥{}^{2}} \end{array}$$

where $t_{c}$ is the converging time step.

An *n*-dimensional column vector comprising of all ones (or zeros) is denoted as $1_{n}$ (or $0_{n}$). $I_{n}$ denotes an n×n identity matrix. The initialization parameters of mPMHE algorithm are listed as follows:

- The initial state vectors $x_{0}^{\left[1\right]}=x_{0}^{\left[4\right]}={\left[\begin{array}{c} 1_{3}^{T},0_{3}^{T} \end{array}\right]}^{T}$; $x_{0}^{\left[2\right]}=x_{0}^{\left[3\right]}={\left[\begin{array}{c} 1_{4}^{T},0_{4}^{T} \end{array}\right]}^{T}$.
- The initial covariance matrices: $P_{1|0}=10^{3}I_{28}$, $\Pi_{0}^{\left[1\right]}=\Pi_{0}^{\left[4\right]}=10^{3}I_{6}$, $\Pi_{0}^{\left[2\right]}=\Pi_{0}^{\left[3\right]}=10^{3}I_{8}$.
- The noise covariances: $Q_{0}^{\left[1\right]}=Q_{0}^{\left[4\right]}=10^{-6}I_{6}$, $Q_{0}^{\left[2\right]}=Q_{0}^{\left[3\right]}=10^{-6}I_{8}$; $R_{0}^{\left[i\right]}\;=\;\mathrm{diag}\left(\sigma_{1}^{2},\ldots ,\sigma_{m_{i}}^{2}\right),i\;=\;1,\ldots ,4$;
- The horizon length: N+1=3.
- State constraints: $0.9\leq {\hat{V}}_{i}^{r}\leq 1.2$, $-0.35\leq {\hat{V}}_{i}^{im}\leq 0.01$, where i=1,…,14.

Two different cases which consider the measurements including Gaussian and non-Gaussian noises are presented as follows:

**Case 1: Measurements with Gaussian noise**

In this case, the measurement noises are assumed to be Gaussian. The standard deviation of the voltage phasor measurement is arbitrary chosen as $\sigma_{i}=0.005,i=1,\ldots ,12$ and that of the current phasor measurements is set as $\sigma_{i}=0.01,i=13,\ldots ,58$, according to [31].

**Figure 3:**
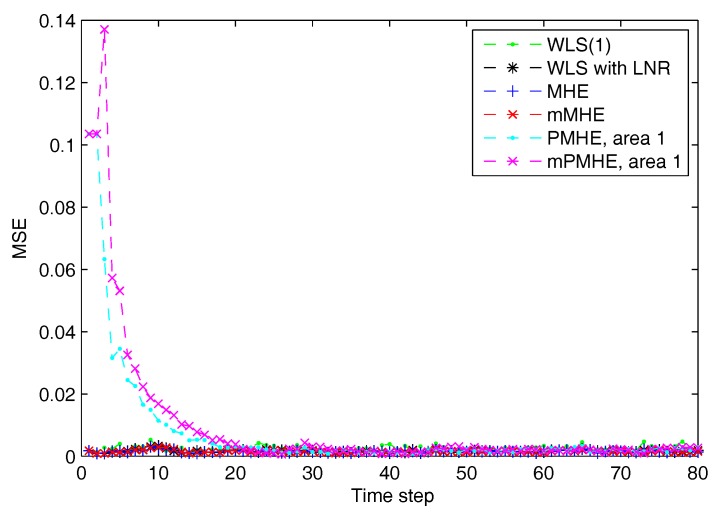
The Mean Square Error (MSE) of WLS(1) (the horizon length of measurements 1), MHE, mMHE, PMHE and mPMHE with constraints, under the assumption of Gaussian noise.

**Figure 4:**
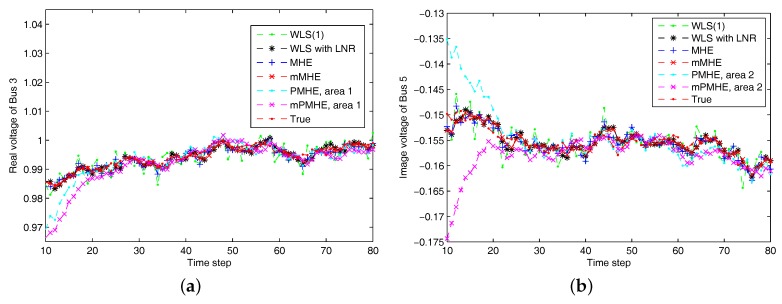
The estimated results of different estimators under Gaussian noise assumption. “WLS(1)” represents the results when the WLS estimator uses the measurements with horizon length 1. (**a**) the real part of Bus 3 voltage phasor. (**b**) the imaginary part of Bus 5 voltage phasor.

The simulations are performed using MATLAB version R2012b on a Windows 10 computer configured with Intel^®^ Core™, CPU i7-4500U, 1.80 GHz and 8 GB RAM, where the quadratic program problems arising from mMHE and mPMHE are solved by the alternating direction method of multipliers (ADMM), following the method presented in [14]. The constraints on the state variables are taken into account both in the MHE, mMHE, the PMHE in [24] and the proposed mPMHE to handle outliers. The comparison of AMSE values obtained from different estimators are given under the “Gaussian” column in Table 2. Even though the WLS (with horizon length 1) spends the least time, 0.3 ms, its AMSE value however is the largest. That is because the WLS estimator does not need to perform any iteration to obtain the estimated results. However, the WLS is not a robust estimator. When the horizon length is set as 3, the WLS takes more time, 1.6 ms, to obtain the estimated results. The AMSE value of WLS with LNR is $1.6\times 10^{-3}$ and it takes 7.7 ms. The LAV estimator is built up according to the method provided by [3] and is conducted based on the matlab subfunction provided in GUROBI example. The AMSE value of LAV is equal to that of WLS with LNR, but the LAV spends more time than WLS with LNR in this case. It is significant to note that the MHE gets the highest accuracy (i.e., having the smallest AMSE $1.2\times 10^{-3}$ ), compared with that of the WLS and WLS with LNR. The AMSE of mMHE with constraints is $1.3\times 10^{-3}$, which is quite close to the result of MHE. However, the mMHE with constraints only takes 6.6 ms while the MHE with constraints takes 11.8 ms. The mMHE takes 44% less time than that of MHE. The traditional PMHE gets better results than the mPMHE, but the mPMHE takes 2.6 ms and it is faster than PMHE, 4.9 ms. The time taken by the has a decreased percentage of 47% compared with PMHE, and a reduction of 60% compared with the centralized mMHE. Even though the mPMHE sacrifices slight estimated accuracy, its computation reduction is significant compared with that of PMHE. Figure 3 shows the convergence of mPMHE with constraints and the converging time step is around 20 time steps. The mPMHE and PMHE finally converges to the centralized results respectively. The convergence rate of mPMHE is quite close to that of PMHE but the computation load reduction of mPMHE is significant. Moreover, the estimated results of some buses from time step 10 to 80 are highlighted in Figure 4 in order to see the difference among different estimators.

**Figure 5:**
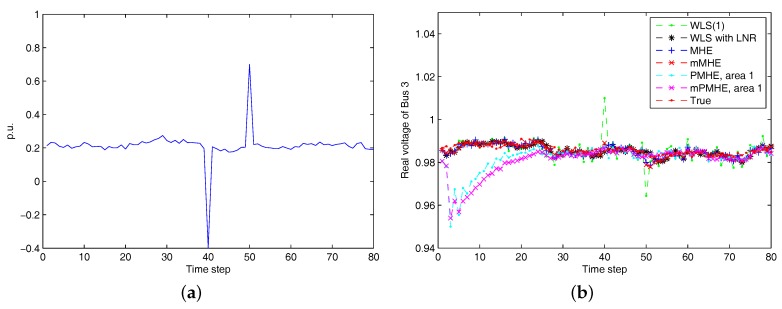
(**a**):The raw current measurement $I_{43}^{r}$ affected by high-magnitude outliers at time steps 40 and 50. (**b**) The estimated result ${\hat{V}}_{3}^{r}$ when outliers occur to measurement $I_{43}^{r}$. “WLS(1)” represents the results when the WLS estimator uses the measurements with horizon length 1.

In order to test the proposed estimator affected by the high-magnitude outliers, suppose the outliers occur in the real part of current measurement $I_{43}^{r}$ at time steps 40 and 50, as shown in Figure 5a. From Figure 5b, we can see that the WLS is seriously affected by the outliers, while the MHE, mMHE, PMHE and mPMHE can deal with the bad data and can still get good estimated results.

**Case 2: Measurements with non-Gaussian noise**

In order to verify that the proposed estimator can also deal with more outliers, the measurement noises are assumed to be non-Gaussian. For the voltage measurements $z_{i,k}$, i=1,…,12, the noise $v_{i}$ is associated with the probability density function as follows:

(21) $$\begin{array}{ccc} f_{i}\left(v_{i}\right) & = & \frac{0.97}{\sqrt{2\pi \sigma_{i}^{2}}}\exp\left(-\frac{v_{i}^{2}}{2\sigma_{i}^{2}}\right)+\frac{0.03}{\sqrt{2\pi {\left(10\sigma_{i}\right)}^{2}}}\exp\left(-\frac{v_{i}^{2}}{2{\left(10\sigma_{i}\right)}^{2}}\right) \end{array}$$

where $\sigma_{i}=0.005$. The first term accounted for 97% in $f_{i}\left(v_{i}\right)$ and the second term having a larger standard deviation is assumed to be the outliers [32].

For the current measurements $z_{i,k}$, i=13,…,58, the noise $v_{i}$ is associated with the probability density function as follows [33]

(22) $$\begin{array}{c} f_{i}\left(v_{i}\right)=\left\{\begin{array}{cc} \frac{0.97}{\sqrt{2\pi \sigma_{i}^{2}}}\exp\left(-\frac{v_{i}^{2}}{2\sigma_{i}^{2}}\right)+\frac{0.03}{2\times 10\sigma_{i}} & |v_{i}|\leq 10\sigma_{j}\\ \frac{0.97}{\sqrt{2\pi \sigma_{i}^{2}}}\exp\left(-\frac{v_{i}^{2}}{2\sigma_{i}^{2}}\right) & \text{otherwise} \end{array}\right. \end{array}$$

where $\sigma_{i}=0.01$. The 3% of uniform distribution in (22) is useful for modeling initial conditions, disturbances and measurement errors that are equally likely to occur anywhere within a given interval.

**Figure 6:**
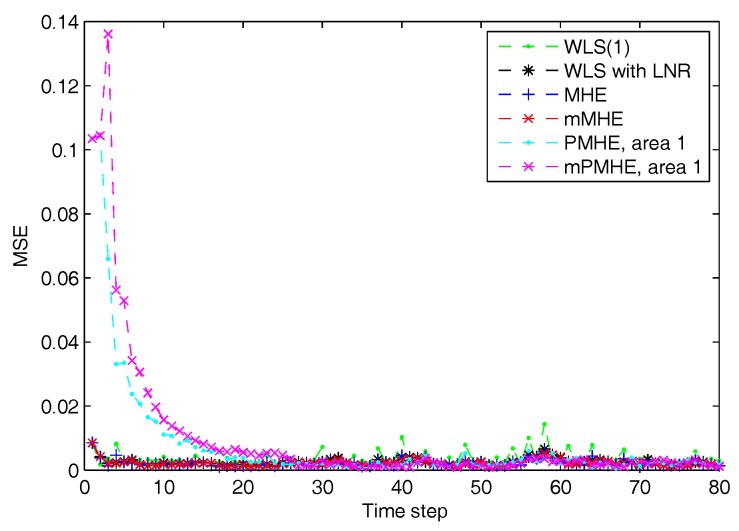
The Mean Square Error (MSE) of WLS(1) (the horizon length of measurements is 1), MHE, mMHE, PMHE and mPMHE with constraints, under the non-Gaussian noise assumption.

**Figure 7:**
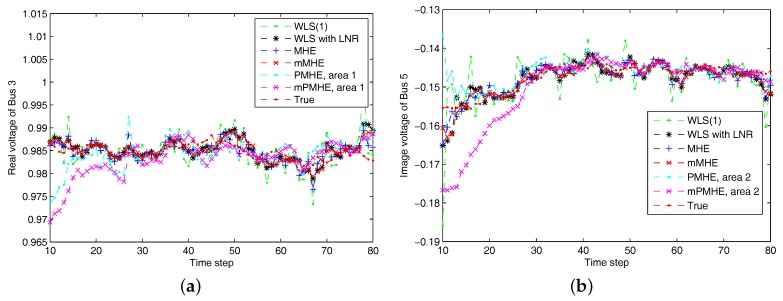
The estimated results of different estimators under non-Gaussian noise assumption. “WLS(1)” represents the results when the WLS estimator uses the measurements with horizon length 1. (**a**) the real part of Bus 3 voltage phasor. (**b**) the imaginary part of Bus 5 voltage phasor.

According to the results shown in Figure 6, both PMHE and mPMHE also converge to the centralized results. However, the MSE is a little larger and it is not as smooth as that shown in Figure 3. That is because many outliers have been included in the measurements. The convergence time seems slightly longer and it is around 30 time steps according to Figure 7. Referring to the AMSE and average time under the “non-Gaussian” column in Table 2, the AMSE values are larger than those under Gaussian assumption, due to the occurrence of outliers. The WLS is not a robust estimator so its AMSE is the highest, $3.7\times 10^{-3}$. The WLS with LNR can detect the bad data, updated the relevant measurements and then the weights of the outliers can be mitigated. More iteration steps are needed for the WLS with LNR so the time increases to 14.0 ms. The AMSE of WLS with LNR is $1.8\times 10^{-3}$. With the inclusion of the constraints, the MHE can handle the outliers and gets the minimum value, $1.7\times 10^{-3}$. The AMSE of the mMHE with constraints is $1.8\times 10^{-3}$, while it only takes 7.3 ms but the MHE takes 15.9 ms to get one set of estimated results. The decreasing percentage of mMHE with constraints is 54%. The AMSE obtained from the distributed approaches finally converge to the centralized results, i.e., $1.7\times 10^{-3}$ for the PMHE with constraints and $1.8\times 10^{-3}$ for the mPMHE with constraints. Their convergence rates are still quite similar. The estimated error of mPMHE is slightly larger than that of PMHE but their difference is insignificant. However, the average computation time of the distributed approach (mPMHE with constraints) is reduced further, 3.7 ms, and it has a reduction of 43% compared with the PMHE with constraints (6.5 ms). The mPMHE with constraints has much bigger reduction percentages, 74% and 69%, compared with the common methods, WLS with LNR (14.0 ms) and LAV (12.0 ms), respectively.

#### 4.1.2. Full Observation with Minimum Number of PMUs

**Figure 8:**
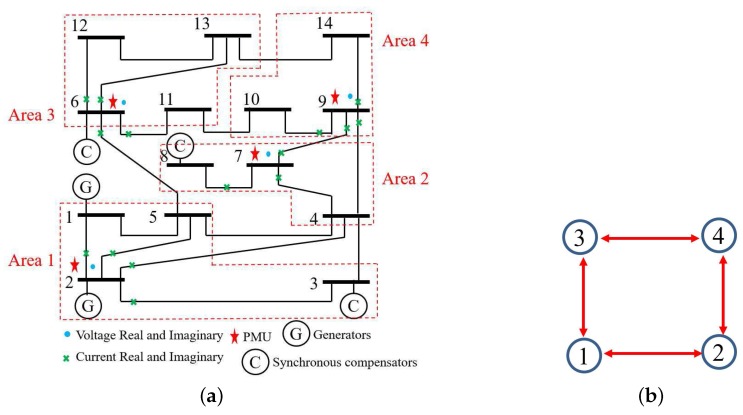
(**a**) The IEEE 14-bus system installed with minimum number of PMUs. (**b**) The communication scheme related to the partitioned type.

In this example, the IEEE 14-bus system has been installed with minimum number of PMUs. If the PMUs are only installed at Buses 2, 6, 7 and 9 according to [26], Assumption 2 is not guaranteed when the local areas is partitioned as the one shown in Figure 1. A new partitioned type is required, as shown in Figure 8a and the relevant communication scheme is shown in Figure 8b.

**Figure 9:**
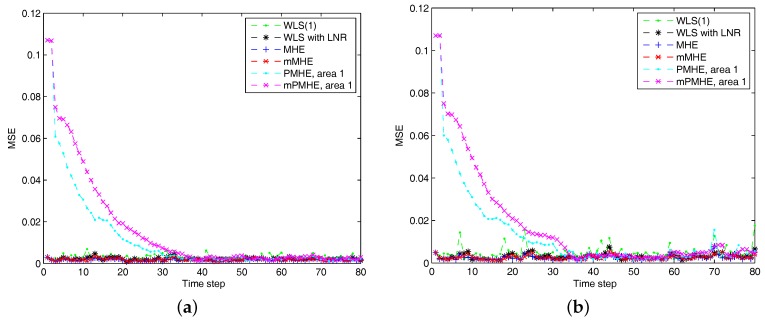
The Mean Square Error (MSE) of different estimators. “WLS(1)” represents the results when the WLS estimator uses the measurements with horizon length 1. (**a**) under Gaussian assumption. (**b**) under non-Gaussian assumption.

A total number of 38 measurements, consisting of eight voltages and 30 currents are taken at each time and the measurements with horizon length 3 are used to give one set of estimates. The standard deviations of the voltage and current measurement noises follow those given in the previous subsection, and the MSE of different estimators under Gaussian and non-Gaussian assumptions are shown in Figure 9a,b respectively. It is significant that the converging time of mPMHE and PMHE with constraints is around 35 time steps. The AMSE and average time are given in Table 3. Due to the smaller number of measurements, the AMSE values are larger than those in Table 2, while the average time is less than those in Table 2. No matter under the Gaussian or non-Gaussian noise assumption, the AMSE values of mPMHE is equal to that of mMHE and a little larger than that of MHE, but the average computation time is much smaller than those for LAV, MHE and mMHE. For example, under the non-Gaussian noise assumption, the average computation time of mPMHE is 2.8 ms, and it has reduction percentages of 47% and 35%, compared with the centralized estimator (LAV, 5.3 ms) and the distributed state estimation method (PMHE, 4.3 ms).

### 4.2. The IEEE 118-Bus System with Non-Gaussian Noise

#### 4.2.1. Redundant Observations

**Figure 10:**
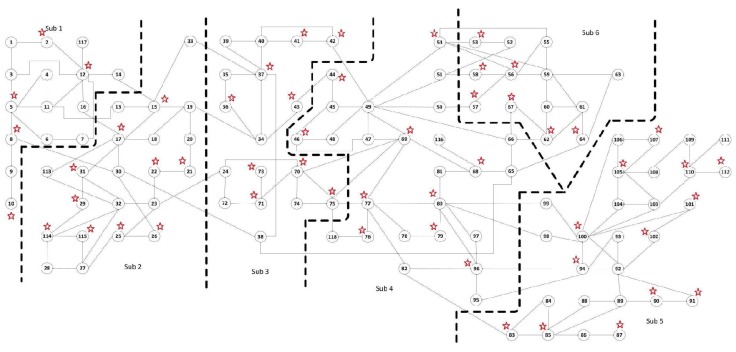
The IEEE 118-bus system installed with 54 PMUs is separated into 6 local areas (subsystems) [34].

**Figure 11:**
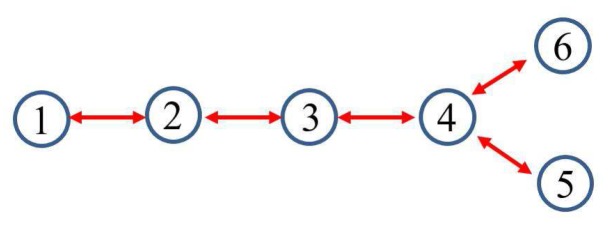
The communication scheme related to the partitioned IEEE 118-bus system.

In order to verify that the proposed algorithm is also effective in large-scale system, the IEEE 118-bus system is used and is partitioned into six local areas (subsystems) as shown in Figure 10. The relevant communication scheme is simplified as shown in Figure 11. The PMUs are placed according to [26] where 54 PMUs are used. A total number of 108 voltage measurements with the noise pdf in (21) with $\sigma_{i}=0.005,i=1,\ldots ,108$ and 366 current measurements with the noise pdf in (22) with $\sigma_{i}=0.01,i=109,\ldots ,474$ are considered. Area 1 has 46 measurements, Area 2 has 78 measurements, Area 3 has 76 measurements, both Area 4 and 5 have 110 measurements respectively, and Area 6 has 54 measurements. Every local area is locally observable. The initialization parameters of mPMHE algorithm in the IEEE 118-bus system are listed as follows:

- The initial state vectors $x_{0}^{\left[1\right]}={\left[\begin{array}{c} 1_{15}^{T},0_{15}^{T} \end{array}\right]}^{T}$; $x_{0}^{\left[2\right]}={\left[\begin{array}{c} 1_{21}^{T},0_{21}^{T} \end{array}\right]}^{T}$; $x_{0}^{\left[3\right]}={\left[\begin{array}{c} 1_{17}^{T},0_{17}^{T} \end{array}\right]}^{T}$; $x_{0}^{\left[4\right]}={\left[\begin{array}{c} 1_{25}^{T},0_{25}^{T} \end{array}\right]}^{T}$; $x_{0}^{\left[5\right]}={\left[\begin{array}{c} 1_{27}^{T},0_{27}^{T} \end{array}\right]}^{T}$; $x_{0}^{\left[6\right]}={\left[\begin{array}{c} 1_{13}^{T},0_{13}^{T} \end{array}\right]}^{T}$.
- The initial covariance matrices: $\Pi_{0}^{\left[1\right]}=10^{3}I_{30}$, $\Pi_{0}^{\left[2\right]}=10^{3}I_{42}$, $\Pi_{0}^{\left[3\right]}=10^{3}I_{34}$, $\Pi_{0}^{\left[4\right]}=10^{3}I_{50}$, $\Pi_{0}^{\left[5\right]}=10^{3}I_{54}$, $\Pi_{0}^{\left[6\right]}=10^{3}I_{26}$.
- The noise covariances: $Q_{0}^{\left[1\right]}=10^{-6}I_{30}$, $Q_{0}^{\left[2\right]}=10^{-6}I_{42}$, $Q_{0}^{\left[3\right]}=10^{-6}I_{34}$, $Q_{0}^{\left[4\right]}=10^{-6}I_{50}$, $Q_{0}^{\left[5\right]}\;=\;10^{-6}I_{54}$, $Q_{0}^{\left[6\right]}=10^{-6}I_{26}$. $R_{0}^{\left[i\right]}=\mathrm{diag}\left(\sigma_{1}^{2},\ldots ,\sigma_{m_{i}}^{2}\right),i=1,\ldots ,6$;
- The horizon length: N+1=3.
- State constraints: $0.85\leq V_{i}^{r}\leq 1.1$, $-0.4\leq V_{i}^{im}\leq 0.4$, where i=1,…,118.

**Figure 12:**
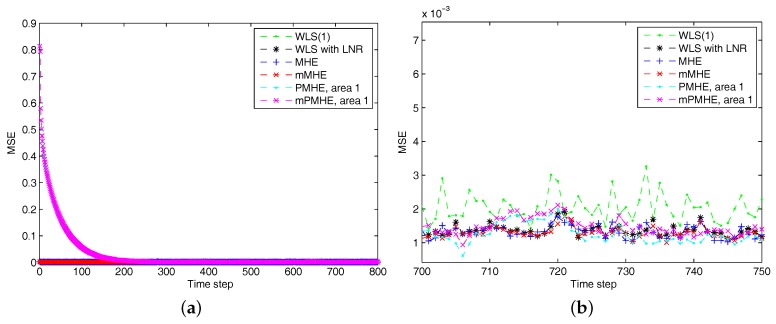
The Mean Square Error (MSE) of WLS(1) (the horizon length of measurements is 1), RLS, MHE, mMHE, PMHE and mPMHE with constraints in the IEEE 118-bus system with redundant observations. (**a**) The MSE from step 1 to 800. (**b**) The details of MSE from step 700 to 750.

**Figure 13:**
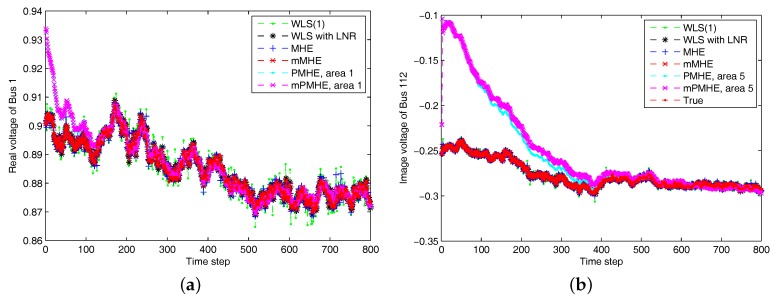
The estimated results of different estimators under non-Gaussian noise assumption. “WLS(1)” represents the results when the WLS estimator uses the measurements with horizon length 1. (**a**) The real part of Bus 1 voltage phasor. (**b**) The imaginary part of Bus 112 voltage phasor.

According to the MSE result shown in Figure 12 and the estimated results shown in Figure 13, it is clear that the estimated results of PMHE and mPMHE converge to the centralized results obtained from MHE and mMHE. The convergence rate of mPMHE is still quite close to that of PMHE and the convergence cannot be expected in fewer than 400 time-steps. The AMSE and average computation time (per step) are given in Table 4. The mMHE still takes less time than that of MHE. The mPMHE with constraints can still get good estimated result and it takes the least time, 32 ms, compared with other robust estimators. The mPMHE reduces about 95% execution time compared with the centralized method (mMHE). Moreover, the mPMHE with constraints has a percentage decrease of 42% compared with that of PMHE (55 ms).

#### 4.2.2. Full Observation with Minimum Number of PMUs

In this case, a minimum number of 32 PMUs are placed in the IEEE 118-bus system according to [26], and the whole system is partitioned into six local areas (subsystems) as shown in Figure 10. In order to make sure that every local area is locally observable, Bus 13 needs to be placed into Area 1 (Sub 1) and Buses 19 and 33 should be returned to Area 3 (Sub 3). The communication scheme is the same as that shown in Figure 11. A total number of 64 voltage measurements with the noise pdf in (21) with $\sigma_{i}=0.005,i=1,\ldots ,64$ and 250 current measurements with the noise pdf in (22) with $\sigma_{i}=0.01,i=65,\ldots ,314$ are considered.

**Figure 14:**
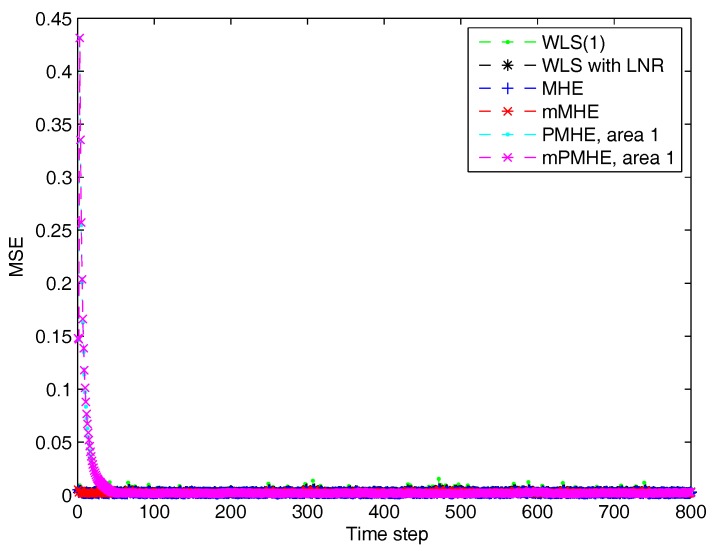
The Mean Square Error (MSE) of WLS(1) (the horizon length of measurements is 1), MHE, mMHE, PMHE and mPMHE with constraints in the IEEE 118-bus system installed with minimum number of PMUs.

According to the MSE result shown in Figure 14, it is clear that the estimated results of PMHE and mPMHE converge to the centralized results obtained from MHE and mMHE. The convergence rate of mPMHE is still quite close to that of PMHE and it is faster than that shown in Figure 12. This verifies that the structure of matrix *H* affects the convergence rate and the details of convergence property can be found in [24]. Compared with those under the redundant observations, the larger AMSE values and smaller average computation time (per step) will be obtained under the “Observation with minimum number of PMUs” column in Table 4. The mPMHE with constraints can still get good estimated result and it reduces about 62% execution time compared with the centralized method (LAV). Moreover, the mPMHE with constraints has a percentage decrease of 36% compared with that of PMHE (33 ms).

According to the simulation results on the IEEE 14-bus and 118-bus systems, given in Table 2 and Table 4, it is clear that the average computation time of mPMHE increases, due to the dimension of local optimization problem in the IEEE 118-bus system which is larger than the IEEE 14-bus system. Therefore, the computation burden depends on the size of partitioned local areas and it would be scalable even when the size of a power system is larger than the IEEE 118-bus system. The computational load can be reduced if the larger power system is partitioned into more local areas (subsystems) and the dimension of each local optimization problem is smaller than that in the IEEE 118-bus system.

## 5. Conclusions

Based on the wide-area monitoring systems, a fully distributed state estimation (mPMHE) based on the moving horizon estimation is proposed for power system state estimation, where each local area solves a smaller optimization problem to estimate its own local states by using the local measurements and the estimated results from its neighboring areas. The computation load is reduced compared with that of the centralized methods. In contrast with PMHE, the error from the process model is ignored in our proposed method. The estimated precision of mPMHE is slightly lower than that of PMHE but their difference is insignificant. The mPMHE achieves comparable state estimation accuracy but with a significant reduction in the computation load.

## Figures and Tables

**Table 1:** Local measurements at time *k* allocated for each local area in the IEEE 14-bus system.

Area	Number of Local Measurements	Measurements
1	10	$V_{2}^{r},V_{2}^{im},I_{1,2}^{r},I_{2,3}^{r},I_{2,4}^{r},I_{2,5}^{r},$
		$I_{1,2}^{im},I_{2,3}^{im},I_{2,4}^{im},I_{2,5}^{im}$
2	20	$V_{4}^{r},V_{7}^{r},V_{4}^{im},V_{7}^{im},I_{4,2}^{r},I_{4,3}^{r}$
		$I_{4,5}^{r},I_{4,7}^{r},I_{4,9}^{r},I_{7,4}^{r},I_{7,8}^{r},I_{7,9}^{r}$
		$I_{4,2}^{im},I_{4,3}^{im},I_{4,5}^{im},I_{4,7}^{im},I_{4,9}^{im},I_{7,4}^{im},I_{7,8}^{im},I_{7,9}^{im}$
3	20	$V_{6}^{r},V_{9}^{r},V_{6}^{im},V_{9}^{im},I_{6,5}^{r},I_{6,11}^{r},$
		$I_{6,12}^{r},I_{6,13}^{r},I_{9,4}^{r},I_{9,7}^{r},I_{9,10}^{r},I_{9,14}^{r}$
		$I_{6,5}^{im},I_{6,11}^{im},I_{6,12}^{im},I_{6,13}^{im},I_{9,4}^{im},I_{9,7}^{im},I_{9,10}^{im},I_{9,14}^{im}$
4	8	$V_{13}^{r},V_{13}^{im},I_{13,6}^{r},I_{13,12}^{r},I_{13,14}^{r},$
		$I_{13,6}^{im},I_{13,12}^{im},I_{13,14}^{im}$

**Table 2:** The Average of Mean Square Error (AMSE) and average computation time (per step) with different estimators in the IEEE 14-bus system with redundant observations.

Noise		Gaussian		Non-Gaussian	
Estimator	Horizon Length	AMSE	Average Time	AMSE	Average Time
		$\left(\times 10^{-3}\right)$	(ms)	$\left(\times 10^{-3}\right)$	(ms)
WLS	1	2.4	0.3	3.7	0.4
	3	1.6	1.6	2.1	1.9
WLS with LNR	3	1.6	7.7	1.8	14.0
LAV	3	1.6	11.3	1.8	12.0
MHE	3	1.2	11.8	1.7	15.9
mMHE	3	1.3	6.6	1.8	7.3
PMHE in [24] (area 1)	3	1.2	4.9	1.7	6.5
PMHE in [24] (area 2)					
PMHE in [24] (area 3)					
PMHE in [24] (area 4)					
mPMHE (area 1)	3	1.3	2.6	1.8	3.7
mPMHE (area 2)					
mPMHE (area 3)					
mPMHE (area 4)					

Remark: The moving horizon estimation (MHE), modified MHE (mMHE), partitioned moving horizon estimation (PMHE) and modified PMHE (mPMHE) take constraints into account during the optimization process.

**Table 3:** The AMSE and average computation time (per step) with different estimators in the IEEE 14-bus system installed with minimum number of PMUs.

Noise		Gaussian		Non-Gaussian	
Estimator	Horizon Length	AMSE	Average Time	AMSE	Average Time
		$\left(\times 10^{-3}\right)$	(ms)	$\left(\times 10^{-3}\right)$	(ms)
WLS	1	3.2	0.2	5.1	0.2
	3	2.0	0.6	4.1	0.7
WLS with LNR	3	2.0	2.3	2.4	3.9
LAV	3	2.0	4.4	2.4	5.3
MHE	3	1.7	5.3	2.1	7.4
mMHE	3	1.8	3.7	2.3	4.9
PMHE in [24] (area 1)	3	3.3	3.3	2.1	4.3
PMHE in [24] (area 2)					
PMHE in [24] (area 3)					
PMHE in [24] (area 4)					
mPMHE (area 1)	3	1.8	2.3	2.3	2.8
mPMHE (area 2)					
mPMHE (area 3)					
mPMHE (area 4)					

Remark: The moving horizon estimation (MHE), modified MHE (mMHE), partitioned moving horizon estimation (PMHE) and modified PMHE (mPMHE) take constraints into account during the optimization process.

**Table 4:** The Average of Mean Square Error (AMSE) and average computation time (per step) with different estimators in the IEEE 118-bus system under two scenarios.

Scenarios		Redundant Observations		Observation with Minimum Number of PMUs	
Number of PMUs		54		32	
Estimator	Horizon Length	AMSE	Average Time	AMSE	Average Time
		$\left(\times 10^{-3}\right)$	(ms)	$\left(\times 10^{-3}\right)$	(ms)
WLS	1	2.1	14	4.2	6.4
	3	1.8	182	2.6	59
WLS with LNR	3	1.4	302	2.2	115
LAV	3	1.4	80	2.2	55
MHE	3	1.3	882	2.1	330
mMHE	3	1.4	669	2.2	264
PMHE in [24] (area 1)	3	1.3	55	2.1	33
PMHE in [24] (area 2)					
PMHE in [24] (area 3)					
PMHE in [24] (area 4)					
mPMHE (area 1)	3	1.4	32	2.2	21
mPMHE (area 2)					
mPMHE (area 3)					
mPMHE (area 4)					

Remark: The moving horizon estimation (MHE), modified MHE (mMHE), partitioned moving horizon estimation (PMHE) and modified PMHE (mPMHE) take constraints into account during the optimization process.

## Abbreviations

The following abbreviations are used in this manuscript:

*m*: Number of measurements at time step *k*
$m_{i}$: Number of local measurements at area *i*
*n*: Number of states
$n_{i}$: Number of local states at area *i*
*x*: True state vector, $x\in R^{n}$
$\hat{x}$: Estimated state vector
$x^{\left[i\right]}$: True local states at area *i*, $x^{\left[i\right]}\in R^{n_{i}}$
$\left({\hat{x}}_{t}^{\left[i\right]}\right)$: Local state vector estimated by the PMHE at time step *t*
$V_{i}^{r}$: Real part of the voltage phasor at bus *i*
$V_{i}^{im}$: Imaginary part of the voltage phasor at bus *i*
$I_{ij}^{r}$: The real part of the current measurement $I_{ij}$
$I_{ij}^{im}$: The imaginary part of the current measurement $I_{ij}$
$w_{k}$: Vector of process noise at time step *k*
$w_{k}^{\left[i\right]}$: Process noise at local area *i* in the PMHE algorithm, $w^{{\left[i\right]}_{k}}\in R^{n_{i}}$
*z*: Measurements from Phasor Measurement Units
*v*: Measurement noise
$e_{i,k}$: The *i*-th measurement residual at time step *k*
$e_{i,k}^{norm}$: The normalized measurement residual *i* at time step *k*
$\rho (e_{i,k})$: Chosen function of $e_{i,k}$
*J*: Cost function
$f_{i}\left(v_{i}\right)$: Probability density function of $v_{i}$
*H*: Measurement matrix
$W_{i,k}$: Weighting factor for *i*-th measurement at time step *k*
Ψ: Derivative of *J* wrt $\hat{x}$
N+1: Horizon length of measurements
*t*, *k*: Time index
$\sigma_{i}$: Standard deviation of measurement noise $v_{i}$
*Q*: Covariance matrix of process noise
*R*: Covariance matrix of measurement noise
*P*: State covariance matrix
X: Constraint set for state *x*
